# Sustained Release Drug Delivery Applications of Polyurethanes

**DOI:** 10.3390/pharmaceutics10020055

**Published:** 2018-05-09

**Authors:** Michael B. Lowinger, Stephanie E. Barrett, Feng Zhang, Robert O. Williams

**Affiliations:** 1 College of Pharmacy, The University of Texas at Austin, 2409 University Avenue, Austin, TX 78712, USA; bill.williams@austin.utexas.edu; 2 MRL, Merck & Co., Inc., 126 E. Lincoln Ave, Rahway, NJ 07065, USA; stephanie_barrett@merck.com

**Keywords:** polyurethane, isocyanate, long-acting, sustained release, drug delivery

## Abstract

Since their introduction over 50 years ago, polyurethanes have been applied to nearly every industry. This review describes applications of polyurethanes to the development of modified release drug delivery. Although drug delivery research leveraging polyurethanes has been ongoing for decades, there has been renewed and substantial interest in the field in recent years. The chemistry of polyurethanes and the mechanisms of drug release from sustained release dosage forms are briefly reviewed. Studies to assess the impact of intrinsic drug properties on release from polyurethane-based formulations are considered. The impact of hydrophilic water swelling polyurethanes on drug diffusivity and release rate is discussed. The role of pore formers in modulating drug release rate is examined. Finally, the value of assessing mechanical properties of the dosage form and approaches taken in the literature are described.

## 1. Introduction

Polyurethanes are among the most ubiquitous of materials found in society, owing to their versatile properties. They can be found in automobiles, chairs, beds, refrigerators and many other household items [1]. Early research into the chemistry of polyurethanes can be found as early as 1947 [2]. By varying different substituents and their ratios, different polyurethanes with a wide range of physicochemical properties can be synthesized at large scales.

This review presents an overview of recent applications of polyurethanes to sustained release drug delivery. Previous review publications generally focused on the chemistry, synthesis and properties of polyurethanes. Cherng et al. authored an extensive review of polyurethane-based drug delivery systems, however it was published over five years ago [3]. Since that time, there has been significant advancement in the research area of polyurethanes, particularly as applied to parenteral sustained release dosage forms.

Polyurethanes have been applied to drug products in nearly every conceivable configuration. Seo and Na explored modifications to polyurethane membrane porosity from a non-erodible drug eluting stent [4]. Guo et al. developed biodegradable polyurethane stent coatings enabling adjustable drug release [5]. Chen et al. explored the use of polyurethane pressure-sensitive adhesives for transdermal drug delivery [6]. Several studies have explored the controlled release of antibiotics from polyurethane matrices through tissue scaffolds [7], bone grafts [8], microspheres [9] and nanoparticles [10]. Temperature- and pH-responsive polyurethane nanoparticles have been developed to deliver doxorubicin to the tumor microenvironment [11]. Drug loaded polyurethane implants have been studied for the treatment of bacterial infection [12] and inflammation [13]. The polymers have been extensively applied in the development of intravaginal rings [14,15,16,17,18,19,20,21,22,23,24,25]. Polyurethanes have also been applied to modulate the release characteristics of orally administered tablets [26]. 

Experimental work exploring monolithic mixtures of a polymer (ethylene vinyl acetate) with model drug compounds has been documented as early as 1964 [27] and the use of polyurethanes in medical devices has been well documented since 1968 [28,29,30,31,32,33,34]. Both biodegradable and biostable sub-dermal implant polyurethane formulations have been of more recent interest [3].

## 2. Chemistry of Polyurethanes

**Figure 1:**
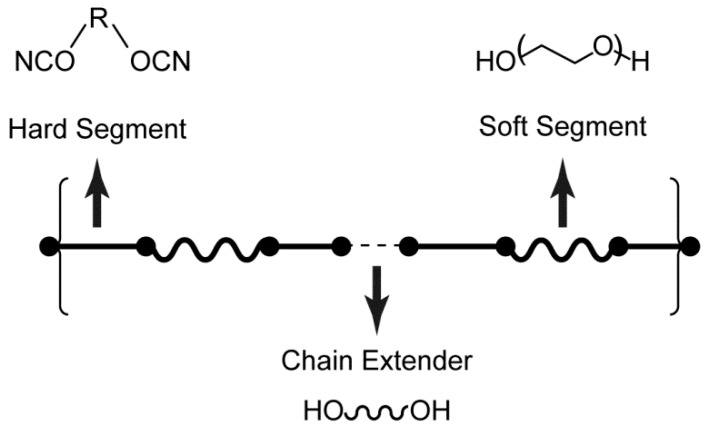
General chemical structure of polyurethanes.

Polyurethanes are a group of condensation polymers that include the urethane (—NHCOO—) group in the chemical structure (Figure 1). They are typically synthesized by a step-growth polymerization reaction between isocyanates and polyols in the presence of a suitable catalyst. Polyurethanes synthesized solely from isocyanates and polyols generally have poor mechanical properties. Therefore, chain extenders are added to the structure in order to induce microphase separation between the two thermodynamically incompatible segments. The two segments are commonly described as hard segments (composed of the isocyanate and chain extender components) and soft segments (composed of the polyol component). The hard segments impart mechanical strength, whereas the soft domains provide flexibility (Figure 1).

### 2.1. Isocyanates

Diisocyanates are commonly employed in the synthesis of polyurethanes, which can be divided into aliphatic and aromatic diisocyanates. In general, aromatic diisocyanates are more reactive than aliphatic species. For example, polyurethanes made from aliphatic diisocyanates demonstrated more resistance to ultraviolet radiation, whereas those made from aromatic diisocyanates have been shown to undergo photodegradation [35,36]. Polyurethanes based on aromatic diisocyanates have also been shown to exhibit less biocompatibility than those synthesized from aliphatic diisocyanates, caused by toxic degradation products. Polyurethanes prepared with toluene diisocyanate have been shown to degrade under physiological conditions to yield 2,4-toluene diamine, which has known toxicity [37]. Kääriä et al. conducted an in vivo study using a polyurethane prepared from the aromatic 4,4′-methylenediphenyl diisocyanate and observed cytotoxicity attributed to the aromatic amine 4,4′-methylenedianiline produced as a degradation product of the polymer [38].

### 2.2. Chain Extenders

Chain extenders are typically low molecular weight (<400 Da) bisamines or diols, such as 1,4-butandiol, 1,3-propanediol and ethylene diamine. The physical and mechanical properties of polyurethanes, including hardness and crystallinity, are dependent on the extent of phase separation between the hard and soft segments. The extent of phase separation is, in part, a function of the type and number of chain extenders used for polymerization. Jabbari and Khakpour investigated the impact of changes to the mole fraction of polyurethane chain extruder to the porosity of prepared microspheres [39]. They observed that the pores in polyurethane microspheres decreased as the content of chain extruder increased from 0 to 50 mol %. When they increased the chain extruder content to 67 mol %, the polymer stiffness increased and formation of pores was inhibited.

### 2.3. Polyols

Polyols are generally di-hydroxyl terminated macroglycols of polyesters, polyethers and polycarbonates in the molecular weight range of 1000 to 5000 Da. The molecular weight and type of polyol plays a significant role in the physicochemical and mechanical properties of the polyurethane. Polyester-based polyurethanes often have good mechanical strength and thermal stability, however they are susceptible to hydrolysis [40]. Biodegradable poly(ester urethanes) have been prepared from lysine diisocyanate with d,l-lactide, ε-caprolactone and other monomers [41]. Kaur et al. developed a biodegradable intravaginal ring composed of a poly(ester urethane) prepared from bis(4-isocynaatocyclohexyl)methane with poly(tetramethylene ether)glycol and ε-caprolactone, which released the antiretroviral dapivirine at target levels for one month [42]. Yu et al. developed biodegradable polyurethanes based on L-phenylalanine that possess tunable mechanical properties and degradation rates over a wider range than was achievable with poly(lactic acid) [43].

Polyether-based polyurethanes tend to be more hydrolytically stable and exhibit more elasticity at lower temperatures. However, they can be more susceptible to oxidative and thermal lability [44,45]. It was found that poly(ether urethane) used as pacemaker lead insulation suffered from stress cracking due to oxidation after being placed in humans for long periods of time [46]. However, antioxidants have been used to stabilize poly(ether urethanes) to prevent oxidation and prolong the life of the polymer [47]. A polyether-based polyol particularly relevant to pharmaceutical applications is polyethylene oxide (PEO). PEO-based polyurethanes exhibit sensitivity to water due to the hydrophilicity and water-absorbing capacity of the ethylene oxide units [3]. Ikeda et al. demonstrated that the larger the PEO content, the higher the degree of swelling which increased the drug release rate of slowly releasing model compounds [48].

Polycarbonate-based polyurethanes were developed in response to the disadvantages of polyester and polyether based polyurethanes. They exhibit good mechanical properties, heat stability and hydrolytic stability but they have been shown to undergo enzymatic hydrolysis and oxidative degradation by inflammatory cells in long-term in vivo studies [49]. 

### 2.4. Synthesis

Polyurethanes are generally synthesized by reacting the isocyanate, polyol and chain extender together at temperatures above 80 °C [50]. The central reaction is the formation of a urethane linkage that occurs when an isocyanate reacts with an alcohol group of the polyol. The exothermic polymerization reaction is generally carried out in one of two ways. The “one-shot method” involves mixing all of the ingredients together, while the “prepolymer method” features the reaction of the polyol with an excess of isocyanate, followed by a subsequent reaction with the chain extender to form a linear block copolymer with alternating blocks of hard segment and soft segment [29]. The prepolymer method has been shown to yield more ordered structure with better control of polymer properties [51].

Two manufacturing methods are typically employed for industrial production: the belt process and the reaction extruder process. During the belt process, all components are mixed using a high efficiency mixing head and the reacting liquid mixture is poured onto a belt, where it solidifies. The solid material is then granulated and may be blended with other components and extruded into pellets. Utilization of a reaction extruder allows for the mixing of prepolymers or all components inside of the extruder, where screw design and temperature can be modified to suit the desired product properties. The urethane reaction is nearly complete by the time the material exits the extruder and uniform pellets may be formed by the use of underwater or strand pelletizers [50].

Since phase separation of polyurethanes is dependent on the temperature and shear conditions during polymerization, the process may have a significant influence on the product properties. Consequently, although two polyurethane batches may start from the same raw materials, their physical properties can be very different [50].

## 3. Drug Release Mechanisms

Solute diffusion, polymer swelling and polymer erosion or degradation are generally considered to be the main driving forces for drug transport from a polymeric matrix [52]. However, other phenomena may be involved in the control of drug release and are discussed in more detail in other publications [53].

### 3.1. Solute Diffusion

Fick’s law of diffusion is the fundamental basis for the mechanism describing drug transport from a polymer matrix. Fickian diffusion refers to a solute transport process in which the polymer relaxation time is much greater than the solvent diffusion time. When polymer swelling occurs, changes to diffusivity with time result in non-Fickian drug release. Drug release from polyurethane formulations can be categorized into two groups: (i) monolithic systems, where drug is dissolved or dispersed in a polyurethane matrix and (ii) reservoir systems, where a drug depot is surrounded by a rate controlling membrane [53]. Table 1 describes the categories of solute diffusion from polyurethane-based sustained release dosage forms.

In each of those categories, drug release kinetics will be dependent on whether the drug concentration is above or below its solubility in the system. In the case of a reservoir system where the initial drug concentration is below its solubility, those drug molecules that diffuse out of the system will not be replaced by undissolved drug and the drug activity at the rate controlling membrane’s surface decreases with time, resulting in first order release kinetics. Models have also been developed which describe first order release kinetics from a cylindrical intravaginal ring [54,55]. However, a reservoir system where the drug concentration exceeds its solubility will feature a saturated solution at the membrane surface, resulting in zero order release kinetics. Over time, drug release kinetics from such a system will approach those of a dosage form with drug concentration below its solubility in the polymer [56].

In the case of monolithic systems, the device geometry and drug loading will significantly affect the drug release kinetics. For a monolithic system where the initial drug concentration is below its solubility in the system, models have been derived to describe the drug release of thin films, spheres and cylinders, many of which assume an exponential function of release rate with time [57,58]. In the case of a monolithic dispersion where the drug is above its solubility in the system, Higuchi described a square root of time relationship between the amount of drug released from a thin film with a large excess of drug [59].

### 3.2. Polymer Swelling

Depending on the polyol used, polyurethanes may exhibit substantial polymer swelling which can impact drug release kinetics in several ways. When a polymer swells, the length of the diffusion pathways increases. This can result in decreasing drug concentration gradients, which may decrease drug release rates. Guo et al. observed that the swelling of a synthesized polyurethane matrix slowed down the drug release rate, which was attributed to increased diffusion length [5]. 

Polymer swelling also increases the mobility of the polymer chains, which increases drug mobility and, potentially, increases drug release rates. Once a water content specific to each polymer is reached, the polymer mobility steeply increases in a phenomenon called “polymer chain relaxation” or “glassy-to-rubbery phase transition” [53]. However, polyurethanes commonly employed for pharmaceutical applications exhibit glass transition temperatures below room temperature, so the transition of polyurethanes from the glassy to the rubbery state is generally not of practical significance to drug release [60]. Clark et al. applied similar pseudo-steady state approach as Higuchi’s diffusion model to effectively predict the release of tenofovir from an intravaginal ring composed of hydrophilic polyurethane [54]. They argued that polymer swelling had minimal impact on the long-term drug release kinetics since the polymers reach equilibrium swelling at early time points and it was thus unnecessary to account for it in the model.

**Figure 2:**
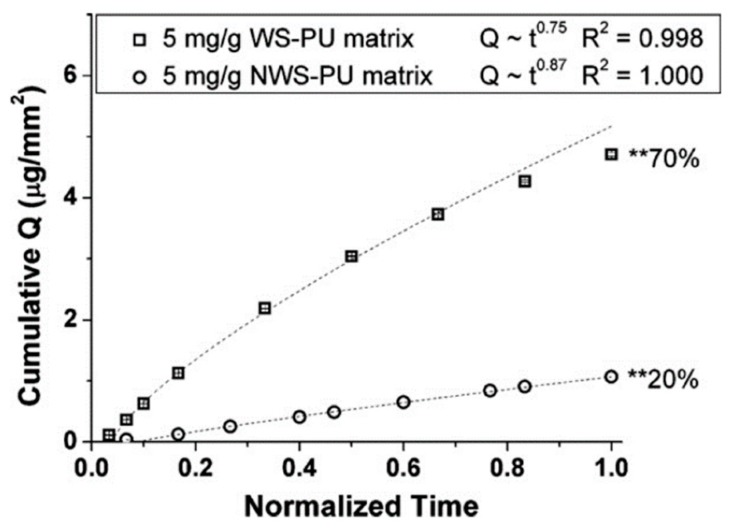
Cumulative flux (Q) of dapivirine as a function of time from a water swelling (WS-PU) and non-water swelling (NWS-PU) polyurethane matrix. ** denotes wt % cumulative release of dapivirine over 30 days. Adapted from [14], Elsevier, 2010 with permission.

Beyond polymer chain mobility itself, water swelling increases free volume for diffusion, thereby increasing diffusivity of drugs [61]. Dapivirine, when released from an intravaginal ring composed of a water-swelling polyurethane grade, exhibited faster release than from a ring composed of non-swelling polyurethane (Figure 2) [14]. Given the wide variety of PEO-based polyurethanes available commercially, polymer swelling has the potential to dramatically impact drug release kinetics from dosage forms.

### 3.3. Polymer Erosion and Degradation

The erosion and degradation of polymers to facilitate drug release are often confounded; however, they will be separated for the purpose of this review. Goepferich and Langer differentiated the two processes by defining degradation as involving cleavage of polymer chains into oligomers and monomers, while erosion can be defined as a general loss of weight from the polymer [62]. Consequently, although degradation of water-insoluble polymers is a step in its erosion process, the degradation of the polymer itself is not erosion. 

Langer and Peppas defined two extremes of erosion: heterogeneous and homogeneous [63]. Heterogeneous erosion describes a physical situation where water penetration into the polymer is slow relative to polymer degradation rate. Under this scenario, polymer degradation is restricted to the outermost layers and erosion predominantly occurs at the surface of the dosage form. In the case of homogeneous erosion, water penetration occurs rapidly, degradation occurs throughout the device and bulk erosion follows. Although all bioerodible polymers are likely to undergo some combination of the two extremes, surface erosion may be most often observed with hydrophobic polyurethanes and those with highly reactive bonds in their backbone structure, whereas hydrophilic polyurethanes and those with less reactive ester linkages are more likely to undergo bulk erosion [53]. Additionally, the water penetration rate may vary depending on the geometry of the delivery system [64]. 

**Figure 3:**
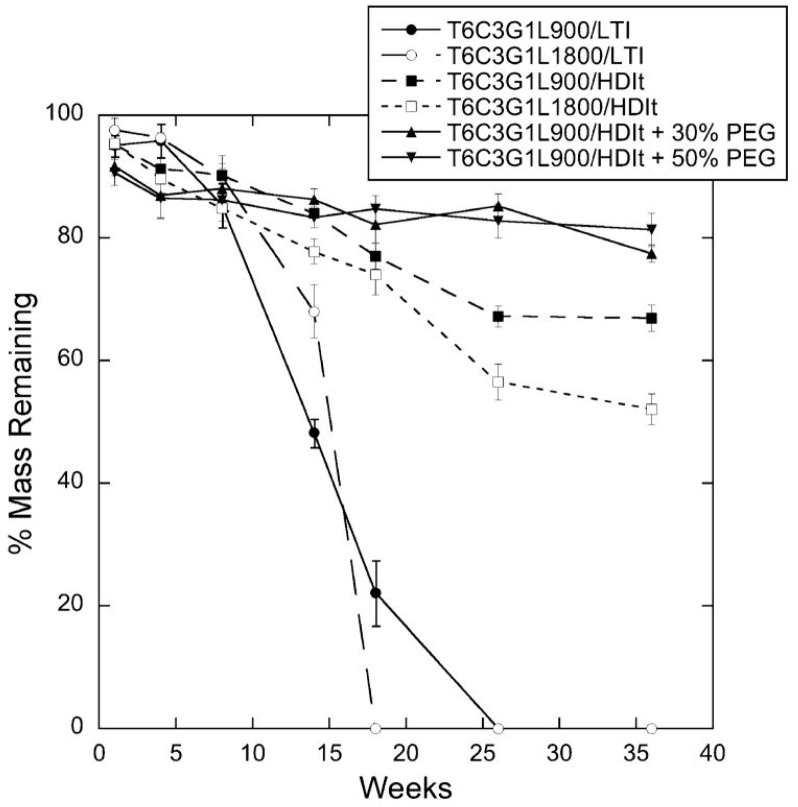
In vitro degradation of polyurethane scaffolds. By 36 weeks, polymers from prepared from lysine triisocyanate (LTI) had completely degraded, while the polyurethanes prepared from hexamethylene diisocyanate trimer remained at 52–81% of their original masses. Adapted from [65], Springer Nature, 2008 with permission.

Hafeman et al. synthesized hydrophilic polyester-based polyurethanes from ε-caprolactone and observed rapid swelling followed by bulk erosion with approximately 50–80% mass remaining after 36 weeks [65] (Figure 3). In a subsequent study investigating the use of one of these polymers to deliver the antibiotic tobramycin, the authors found that the hydrophilic drug released from the polyurethane scaffold over the course of approximately 30 days. Given the difference in time scales between the drug release and polymer degradation rates, the investigators concluded that tobramycin release was independent of polymer degradation [66]. The study demonstrates the ability to develop biodegradable sustained release dosage forms in which drug release kinetics are not dependent on polymer degradation kinetics.

## 4. Approaches to Modulate Drug Release Kinetics

Hombreiro-Pérez et al. described the key mass transport phenomena governing drug release through a polymer, including drug dissolution in the polymer; drug diffusion through the polymer matrix and/or through water-filled pores; drug diffusion through the unstirred liquid boundary layer on the surface of the dosage form; and diffusional and convective transport within the release medium [67]. Through deliberate polymer and formulation selection, the release kinetics of a particular drug may be modulated to achieve a target dose. Table 2 summarizes the approaches that may be taken to modulate drug release kinetics from a polyurethane-based reservoir sustained release dosage form.

### 4.1. Intrinsic Drivers of Drug Release through a Polymer

#### 4.1.1. Drug Solubility in Polymer

In matrix systems where the drug is above its percolation threshold, it is conceivable for drug release to occur by diffusion through drug-rich channels [27,78]. However, for matrix systems where drug load is below its percolation threshold and for all reservoir systems, drug must first dissolve in the polymer in order to diffuse through it. For those formulations, drug solubility in the polymer is an important phenomenon. Johnson et al. found that release of hydrophilic tenofovir with a calculated logP of −2.3 was barely detectable from the hydrophobic polyurethane Tecoflex EG-85A, attributed to poor solubility in the polymer [14]. However, dapivirine with a calculated logP of 6.3 exhibited near zero order release from a similarly hydrophobic polyurethane Tecoflex EG-80A [17]. Van Laarhoven et al. measured the solubility of etonogestrel and ethinyl estradiol in ethylene vinyl acetate copolymers and found that the two hydrophobic drugs were sufficiently soluble in the hydrophobic polymer that they were present in the finished product in a molecularly dissolved state [56]. Clark et al. determined the solubility of tenofovir in a hydrophilic polyurethane and observed that its solubility was 100 to 1000 times lower than the drug loading explored in their studies [54].

#### 4.1.2. Drug Diffusivity through Polymer

**Figure 4:**
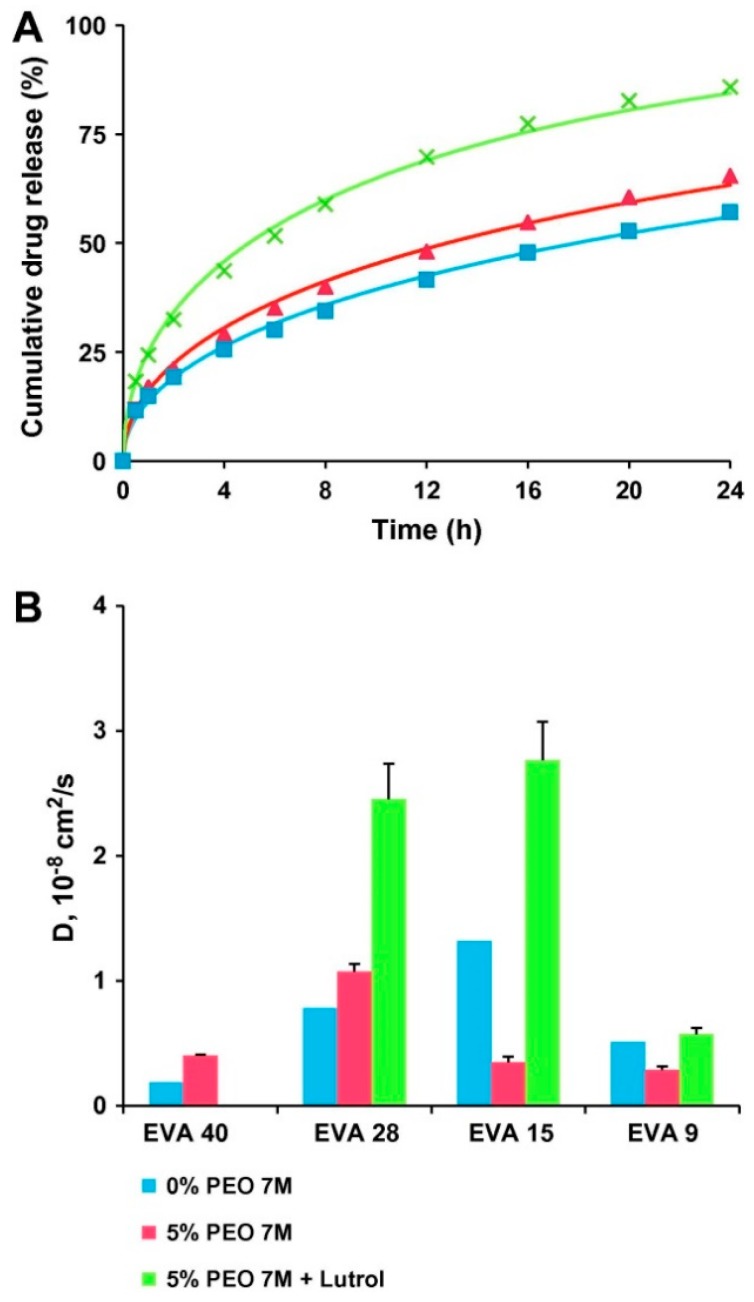
(**A**) Theory (curves) and experiments (symbols): metoprolol tartrate release from EVA 28-based matrices containing 0% PEO 7 M (■), 5% PEO 7 M (▲), or 5% PEG 7 M/Lutrol (9/1, *w*/*w*) (×). (**B**) Apparent diffusion coefficients of metoprolol tartrate in EVA-based matrices, containing 0% PEO 7 M, 5% PEO 7 M, or 5% PEO 7 M/Lutrol (9/1, *w*/*w*). Adapted from [68], Elsevier, 2012 with permission.

The phase state of the polymer has been shown to impact diffusivity of drug through it. Almeida et al. studied the impact of vinyl acetate content on the release rate of metoprolol tartrate from melt extruded ethylene vinyl acetate matrices in the presence of varying levels of polyethylene oxide. Lower vinyl acetate content results in greater crystallinity of the polymer. They found that matrices extruded with lower vinyl acetate content polymers exhibited slower drug release rates than those extruded with higher vinyl acetate content polymers. By fitting the experimental data to an analytical model of Fick’s second law of diffusion, they were able to show that release rate differences between polymers could be explained by changes to the apparent diffusion coefficient (Figure 4) [68]. Tallury et al. explored the impact of ethylene vinyl acetate copolymer composition on the release of chlorhexidine and acyclovir from polymer matrices. They observed a strong relationship between vinyl acetate content and drug release for both systems, where higher vinyl acetate content exhibited faster drug release [69]. Although the effect of polymer crystallinity on drug release from nonerodible polyurethane-based dosage forms has not been extensively studied, several investigators correlated the crystallinity of the soft segment to degradation rate of poly(ester urethanes). Reddy et al. proposed that higher crystallinity of the poly(caprolactone) soft segment resulted in reduced polymer degradation rates, which slowed the release of the model drug theophylline [70]. 

The molecular weight of the polymer may also impact the diffusion of drug through the dosage form. Hsu and Langer investigated the impact of changes to ethylene vinyl acetate molecular weight on the release rate of bovine serum albumin (BSA). They observed a substantial decrease in BSA release rate with relatively small increases in ethylene vinyl acetate molecular weight [71]. Skarja and Woodhouse investigated the effect of molecular weight on the properties of polyurethanes composed of either poly(caprolactone) or poly(ethylene oxide) as the soft segment. They found that phase separation between the hard and soft segments and crystallinity of the soft segment increases with soft segment molecular weight. For polyurethanes based on hydrophobic poly(caprolactone), one might expect reduced drug release rates from a higher molecular weight polymer, however those based on hydrophilic poly(ethylene oxide) might be expected to release drug at faster rates [72].

For polyurethanes, the ratio between soft segment and hard segment has also been shown to affect drug release kinetics. Shoaib et al. explored the effect of soft segment to hard segment ratio on the release of ciprofloxacin. The polyurethane-urea elastomers were synthesized from the aromatic toluene diisocyanate and the hydrophilic polyethylene glycol. As soft segment to hard segment ratio was decreased, the investigators observed a decrease in ciprofloxacin release rate from drug/polymer films. The authors attributed the slower drug release to increased cross-linking of the hard segments in polymers featuring a higher concentration of hard segment. They speculated that increased cross-linking would reduce water penetration into the matrix and drug diffusion out of the matrix [73]. 

**Figure 5:**
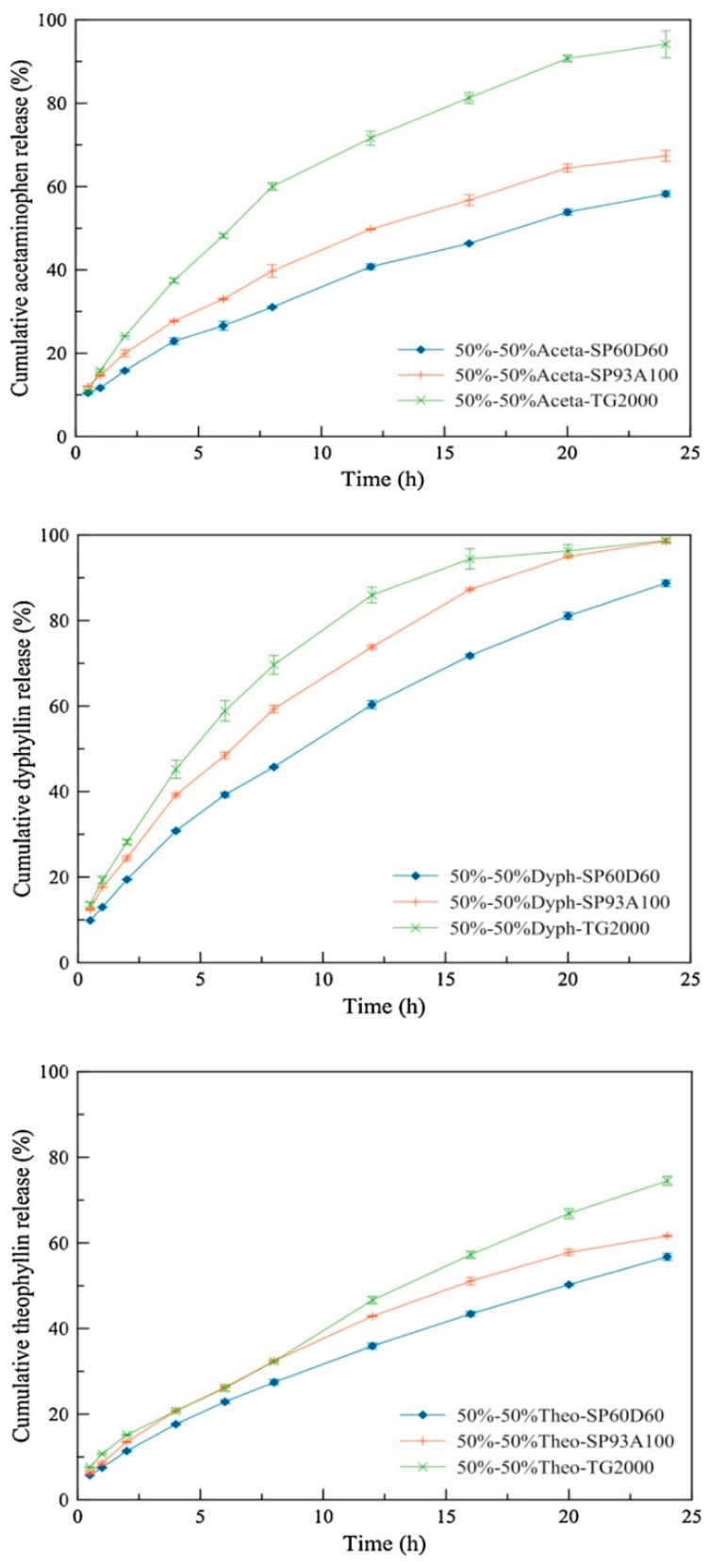
Influence of length of the polyurethane soft segment (polyethylene oxide) on the in vitro release kinetics of drugs with different aqueous solubility (acetaminophen, diprophylline and theophylline) from polyurethane-based matrices (SP60D60, SP93A100 and TG2000). Adapted from reference [74], Elsevier, 2016 with permission.

Verstraete et al. investigated the impact of soft segment to hard segment ratio on the release rates of diprophylline, theophylline and acetaminophen for hydrophilic thermoplastic polyurethanes for which the soft segment is composed of polyethylene oxide. As the soft segment to hard segment ratio increased, the fraction of polyethylene oxide in the polymer structure increased. The authors observed an increase in swelling for the polymers Tecophilic SP60D60, SP93A100 and TG2000 ranging from 60% to 900% weight gain. When investigating the drug release kinetics of the three drug compounds from matrices of each polymer, they found that all drugs followed the same trend with the TG2000-based matrix releasing fastest and the SP60D60-based matrix releasing slowest (Figure 5) [74]. Increased water uptake and faster drug release may be due to the formation of a water-filled pore structure or due to higher free volume that increases diffusivity.

### 4.2. The Use of Pore Formers

The incorporation of soluble components to an otherwise poorly soluble barrier has been utilized as an approach to modulate the release of drugs through film coated tablets for decades [79,80,81,82,83]. A similar approach has been applied to the development of drug/polyurethane dosage forms in order to optimize the drug release rate. Kim et al. evaluated the effect of polyethylene glycol, D-mannitol and bovine serum albumin on the release of the antibiotic cefadroxil from a polyurethane matrix [75]. They observed that matrices utilizing bovine serum albumin as the pore former exhibited the fastest drug release. The authors proposed that immiscibility of the pore former with the polyurethane could facilitate channel formation and thus increase drug release rate. 

**Figure 6:**
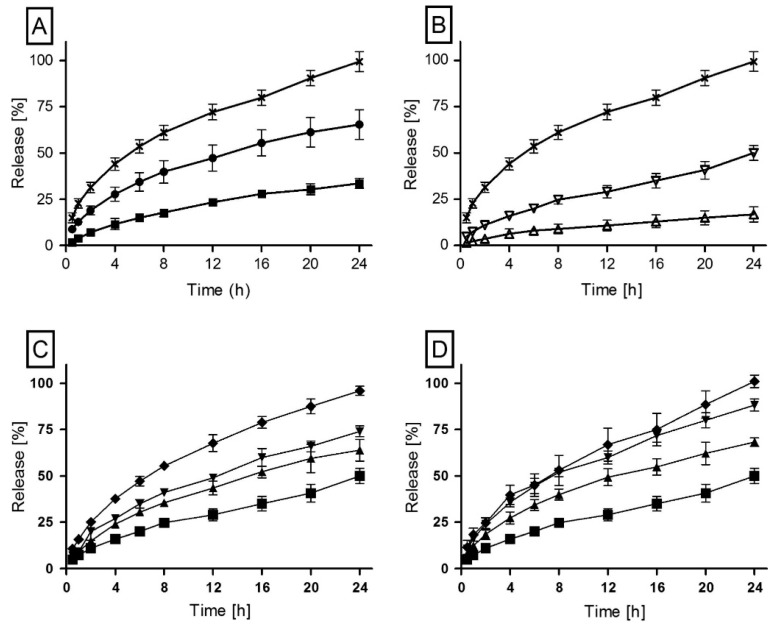
Mean dissolution profiles (±SD) of polyester-based polyurethane (Pearlbond) matrices as a function of (**A**) drug load: 50% (■), 60% (●) and 65% (✕) metoprolol tartrate; (**B**) drug solubility: 65 wt % theophylline (△), diprophylline (▽) and metoprolol tartrate (✕); pore former (**C**) PEG 4000 or (**D**) Polysorbate 80; 65 wt % diprophylline with 0% (■), 2% (▲), 5% (▼) and 10% (◆) of pore former, respectively. Adapted from [60], Elsevier, 2015 with permission.

Donelli et al. investigated the utility of incorporating polyethylene glycol and bovine serum albumin into a polyurethane matrix to modify the release rate of the antifungal drug fluconazole [76]. They found that matrices incorporating polyethylene glycol exhibited increased drug release relative to a control without pore former, whereas matrices incorporating bovine serum albumin exhibited sustained drug release relative to the control. Sreenivasan observed an increased release rate of the anti-inflammatory drug hydrocortisone when adding methyl β-cyclodextrin to polyurethane [77]. Claeys et al. explored the impact of polyethylene glycol, polysorbate 80 and the dicarboxylic acids malonic, succinic, maleic and glutaric acid on the release of diprophylline from a polyurethane matrix (Figure 6) [26,60]. 

## 5. Mechanical Properties of Polyurethane-Based Dosage Forms

Since many polyurethane-based sustained release dosage forms are intended to remain in vivo for extended periods of time, their mechanical properties are critical to ensure consistent drug release kinetics and good patient adherence. The dosage forms must exhibit enough elasticity to deform seamlessly without causing discomfort to patients during routine daily activities and without causing tissue damage or inflammation [84]. On the other hand, the dosage forms must have sufficient strength to prevent fracture, which would alter geometry and potentially affect drug release rate. For example, intravaginal rings that are too soft may not be effectively retained and could be expelled from the vagina [85]. 

### 5.1. Patient Perceptions

Given that many of the mechanical properties are driven by patient perceptions, it can be difficult to determine an appropriate target for a dosage form under development. The target mechanical properties of each dosage form may be dependent on the route of administration and duration of the product. However, patients are likely to have more interaction with intravaginal rings than most other parenteral formulations and therefore investigators have evaluated the mechanical performance of intravaginal rings more extensively than most other presentations. 

Morrow Guthrie et al. conducted a clinical study to understand the relationship between user perceptions and mechanical properties of intravaginal rings composed of polyurethane [86]. Users perceived a ring with a matte and textured surface to be easier to manipulate during insertion, whereas they perceived a ring with a glossy and smooth surface to be slicker and more challenging to insert. The study participants also expected rings composed of softer materials to be more comfortable to use. Although the participants preferred a small diameter ring, it conflicted with their general desire for a more pliable dosage form. For a given material at a defined cylinder diameter, a smaller diameter ring will be more difficult to squeeze. Faced with these tradeoffs, users were more comfortable with using softer materials and smaller diameter cylinders, even if the ring diameter were larger.

### 5.2. Mechanical Testing of Finished Product

Since most investigators lack the clinical data necessary to quantify patient preferences, studies describing the assessment of an investigational dosage form’s mechanical properties typically reference their findings back to a marketed product. Baum et al. proposed several techniques to evaluate the mechanical properties of an experimental silicone-based intravaginal ring, comparing it to the commercially available Estring^®^ [87]. The tensile strength, elongation and compression strength were determined using methods adapted from ASTM D2240 and ISO 8009 standards [88,89].

Verstraete et al. built on Baum’s efforts by applying those techniques to polyurethane-based intravaginal dosage forms and comparing back to the marketed product Nuvaring^®^ [15]. Shore durometer hardness was measured using an indentation test on the surface of the ring. Elongation and force at maximum extension were measured using an extension testing system. To evaluate elongation, a sample was fixed between two hooks and its axial length was measured after applying a defined force. In order to assess maximum elongation, the sample was stretched at a defined rate until breakage. The researchers sought to evaluate resistance to compression by subjecting a sample to repeated compression cycles at a defined speed and amplitude and assessing changes to the diameter along the axis of compression and orthogonal to it. Table 3 provides a summary of the measured intravaginal ring mechanical properties in comparison to the marketed product. By performing a variety of compression, elongation and indentation tests, the investigators were able to assess the mechanical properties of the dosage form under a variety of circumstances.

Clark et al. performed a destructive extension test on their segmented intravaginal ring samples both before and after 31-day in vitro release testing. Samples were stretched at a defined rate until failure was observed at which point the net extension and net load were recorded [90]. The investigators did not compare measured properties back to a marketed product. Without a benchmark, it can be difficult to interpret the outcome as to whether the failure conditions were beyond what is reasonably expected during normal handling. 

Young’s modulus measures a material’s resistance to being deformed elastically when a stress is applied to it. A stiffer material will have a higher Young’s modulus. Ugaonkar et al. leveraged the Young’s modulus as a measure of flexibility by subjecting a 25 mm long cylindrical segment to a defined elongation at a specified rate and again compared the measured value for their experimental dosage form to that of the marketed product Nuvaring^®^ [55]. Clark et al. developed a model to predict the force necessary to compress an intravaginal ring material a given distance based on the Young’s modulus [54]. Although Young’s modulus is an effective measure of stiffness, it does not provide information on elongation and compression properties.

Crnich et al. were interested in understanding the effect of ethanol exposure on the mechanical properties of polyurethane stents. They performed tensile strength testing, including force-at-break, failure stress, elongation at failure, maximum strain and modulus of elasticity in accordance with ISO standard 10555-1 [91]. The investigators concluded that exposure to ethanol had a minimal effect on the mechanical properties of polyurethane catheters.

**Figure 7:**
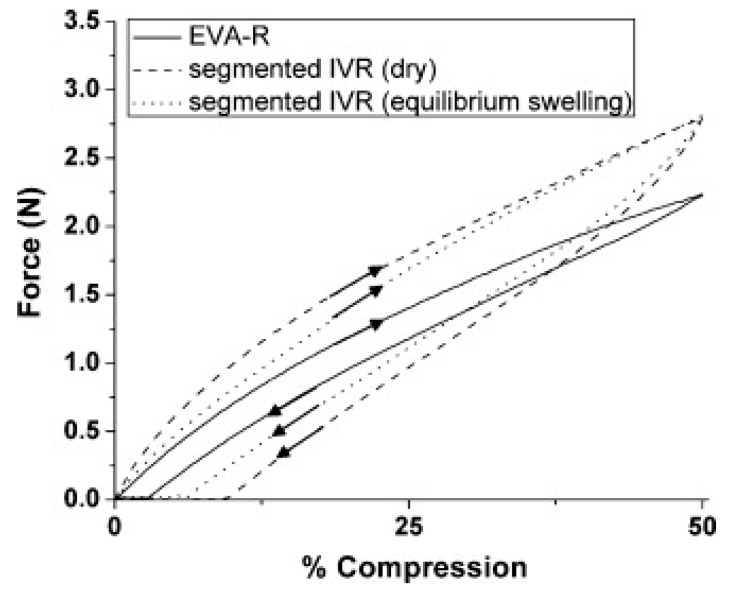
Force versus percent ring compression for experimental (segmented IVR) and Nuvaring^®^ (EVA-R) intravaginal rings. Equilibrium swelling, as determined by mass change, was achieved after 3 days in water. Each ring was brought to 50% compression and subsequently allowed to recover to its original diameter as indicated by the direction of arrows. Adapted from [14], Elsevier, 2010 with permission.

Johnson et al. performed a tensile strength test on their intravaginal ring samples in a similar fashion to others [14]. Rings were stretched to a defined force at a specific rate and any evidence of failure or changes to diameter were assessed. The investigators also performed compression/retraction force tests in which the rings were compressed at a defined rate to 50% of their initial diameter and force was recorded throughout the experiment (Figure 7). They benchmarked to a marketed product and observed that their hydrophilic polyurethane-based intravaginal ring exhibited similar mechanical properties to the Nuvaring^®^ reference (“EVA-R”) when kept dry. However, the hydrated polyurethane-based ring exhibited faster recoil than the reference, which the authors pointed out could improve retention in the vaginal tract.

### 5.3. Gamma Irradiation

Gamma irradiation is an established approach to sterilize materials for biomedical application. However, polymer irradiation may result in crosslinking or chain scission, resulting in physical and mechanical changes to the polymer. It has been generally reported that medical polyurethane products are able to withstand multiple exposures to gamma irradiation without change to physical or mechanical properties [92]. For example, Abraham et al. studied the effect of gamma irradiation on the mechanical properties of an aromatic poly(ether urethane urea) and an aliphatic polycarbonate-based polyurethane [93]. The investigators assessed tensile properties with uniaxial stress-strain data following ASTM D638 methods and elongation properties using a stress hysteresis test. Although they observed a change in molecular weight distribution and soft segment glass transition temperature for both polymers, they found no significant effect of irradiation on the tensile properties and a small increase in hysteresis stress values.

In a separate study, Simmons et al. examined the effect of gamma irradiation on the mechanical properties of an aromatic poly(ether urethane) and an aromatic polyurethane based on both polyether and polysiloxane in the soft segment [94]. They determined the ultimate tensile strength, ultimate elongation and Young’s modulus prior to and following sterilization. Gamma irradiation appeared to stiffen the polyether/polysiloxane-based material, with an approximate 20% increase in Young’s modulus, while tensile strength and elongation remained largely unchanged. There was no significant effect of irradiation on the measured mechanical properties of the polyether-based polyurethane.

Gorna et al. noted that previous studies had focused on the impact of gamma irradiation on the properties of non-erodible polyurethanes. Therefore, they investigated the effect of gamma irradiation on the mechanical properties of biodegradable polyurethanes based on poly(ethylene oxide) and poly(ε-caprolactone), used for medical implants and scaffolds [95]. They measured the tensile strength, Young’s modulus and elongation at break before and after gamma irradiation. The investigators observed a decrease in mechanical strength following gamma irradiation, with the polyurethane based on poly(ethylene oxide) exhibiting a substantial 50% decrease in tensile strength.

However, Ahmed et al. studied the effect of gamma irradiation on the mechanical properties of a non-erodible aromatic poly(carbonate urea) based polyurethane alongside a biodegradable aliphatic polycaprolactone based polyurethane [96]. They observed an approximate 25% decrease in Young’s modulus and ultimate tensile strength for both polymers following irradiation. Consequently, no generalized conclusions can be made with regard to the effect of gamma irradiation on the mechanical properties of varying types of polyurethanes, underscoring the importance of verifying mechanical properties of the drug product during development.

## 6. Conclusions

Owing to their chemical diversity, polyurethanes can be tailored to exhibit a wide variety of physical properties. Crystallinity, hydrophilicity, hydrated porosity, mechanical strength and bioerodibility can be tuned to achieve the desired dosage form characteristics and release rate for a diverse array of treatment duration and route of administration. The diversity of polyurethane chemistry suggests that one may have substantially more degrees of freedom to select a polymer exhibiting good or poor drug solubility than with silicone or poly(ethylene-co-vinyl acetate) elastomers. The ability to tune the extent of water swelling by changing the soft segment to hard segment ratio of polyurethanes presents an exciting route to modulate drug release kinetics independent of drug solubility in polymer. Despite the available opportunities, few commercialized drug products leverage polyurethanes, suggesting that it remains a nascent field with much to be understood before it can be routinely reduced to practice. Polyurethane-based parenteral sustained release dosage forms are well suited toward therapies where high adherence to a consistent dose over a long duration is critical, particularly infectious and neurodegenerative diseases.

## Figures and Tables

**Table 1:** Categories of Solute Diffusion from Polyurethane-based Sustained Release Dosage Forms.

Dosage Form Type	Drug Concentration in Polymer	Release Kinetics	Examples
Monolithic	C_drug_ ≤ C_solubility_	Geometry and drug load dependent	[57,58]
	C_drug_ > C_solubility_	Geometry and drug load dependent	[59]
Reservoir	C_drug_ ≤ C_solubility_	First order	[54,55]
	C_drug_ > C_solubility_	Zero order	[56]

**Table 2:** Approaches to Modulate Drug Release Kinetics from a Polyurethane-based Reservoir Sustained Release Dosage Form.

Driver	Approach	Examples
Drug Solubility in Polymer	Polymer selection to increase or reduce drug solubility	[14,17,54,56]
Drug Diffusivity Through Polymer	Polymer selection to increase or reduce polymer crystallinity	[68,69,70]
	Polymer selection to increase or reduce polymer molecular weight	[71,72]
	Polymer selection to increase or reduce soft segment to hard segment ratio	[73,74]
Drug Diffusion Through Water-filled Channels	Polymer selection to increase or reduce soft segment to hard segment ratio	[73,74]
	Incorporation of additional component as pore former	[75,76,77]

**Table 3:** Overview of intravaginal ring properties (mean ± SD, *n* = 3). Devices that featured similar mechanical properties to reference were highlighted in grey. Adapted from [15], Elsevier, 2017 with permission.

Formulation	Hardness ^a^ (shore A)	Max. Load ^a^ (N)	Max. Elongation ^b^ (%)	OD_1_′/OD_1_^c^ (%)	OD_2_′/OD_2_^c^ (%)
**Reference**					
Nuvaring™	75 ± 4	102.4 ± 12.7	650.1 ± 11.8	92.1	107.5
**Treatment**					
25/75 metronidazole/SP-93-100	72 ± 3	82.8 ± 13.7	587.9 ± 117.4	94.6	104.6
50/50 metronidazole/SP-93-100	91 ± 2	68.1 ± 10.2	51.7 ± 21.4	88.3	110.2
Prophylaxis					
20/80 Lactic Acid/EG-80A	51 ± 1	49.7 ± 12.4	517.0 ± 4.9	98.0	101.7
20/80 Lactic Acid/EG-85A	62 ± 2	68.6 ± 22.7	389.4 ± 34.3	98.2	101.3
20/80 Lactic Acid/EG-93A	71 ± 2	87.7 ± 8.15	336.7 ± 24.9	96.0	103.8
20/80 Lactic Acid/EG-100A	80 ± 2	98.6 ± 11.6	244.6 ± 37.4	94.3	105.1
20/80 Lactic Acid/EG-60D	80 ± 4	105.4 ± 13.8	173.8 ± 22.2	93.7	107.2
20/80 Lactic Acid/EG-72D	86.3 ± 3	129.3 ± 14.1	125.7 ± 13.9	89.5	110.0

^a^ Hardness and maximum load should be similar to the Nuvaring^TM^ reference values. ^b^ Mean elongation at break should not be less than 300%. ^c^ After compression experiments, the diameter along the axis of compression (OD_1_′) and the diameter orthogonal to the axis of compression (OD_2_′) should be at least 90% of their initial values.

## References

[1] Engels H.-W., Pirkl H.-G., Albers R., Albach R.W., Krause J., Hoffmann A., Casselmann H., Dormish J. (2013). Polyurethanes: Versatile Materials and Sustainable Problem Solvers for Today’s Challenges. Angew. Chem. Int. Ed..

[2] Bayer O. (1947). Das Di-Isocyanat-Polyadditionsverfahren (Polyurethane). Angew. Chem..

[3] Cherng J.Y., Hou T.Y., Shih M.F., Talsma H., Hennink W.E. (2013). Polyurethane-based drug delivery systems. Int. J. Pharm..

[4] Seo E., Na K. (2014). Polyurethane membrane with porous surface for controlled drug release in drug eluting stent. Biomater. Res..

[5] Guo Q., Knight P.T., Mather P.T. (2009). Tailored drug release from biodegradable stent coatings based on hybrid polyurethanes. J. Control. Release.

[6] Chen X., Liu W., Zhao Y., Jiang L., Xu H., Yang X. (2009). Preparation and characterization of PEG-modified polyurethane pressure-sensitive adhesives for transdermal drug delivery. Drug Dev. Ind. Pharm..

[7] Li B., Brown K.V., Wenke J.C., Guelcher S.A. (2010). Sustained release of vancomycin from polyurethane scaffolds inhibits infection of bone wounds in a rat femoral segmental defect model. J. Control. Release.

[8] Gorna K., Gogolewski S. (2003). Preparation, degradation, and calcification of biodegradable polyurethane foams for bone graft substitutes. J. Biomed. Mater. Res..

[9] Li B., Yoshii T., Hafeman A.E., Nyman J.S., Wenke J.C., Guelcher S.A. (2009). The effects of rhBMP-2 released from biodegradable polyurethane/microsphere composite scaffolds on new bone formation in rat femora. Biomaterials.

[10] Martinelli A., D’Ilario L., Francolini I., Piozzi A. (2011). Water state effect on drug release from an antibiotic loaded polyurethane matrix containing albumin nanoparticles. Int. J. Pharm..

[11] Wang A., Gao H., Sun Y., Sun Y., Yang Y.-W., Wu G., Wang Y., Fan Y., Ma J. (2013). Temperature- and pH-responsive nanoparticles of biocompatible polyurethanes for doxorubicin delivery. Int. J. Pharm..

[12] Basak P., Adhikari B., Banerjee I., Maiti T.K. (2009). Sustained release of antibiotic from polyurethane coated implant materials. J. Mater. Sci. Mater. Med..

[13] Moura S.A.L., Lima L.D.C., Andrade S.P., Silva-Cunha Junior A. Da, Órefice R.L., Ayres E., Da Silva G.R. (2011). Local Drug Delivery System: Inhibition of Inflammatory Angiogenesis in a Murine Sponge Model by Dexamethasone-Loaded Polyurethane Implants. J. Pharm. Sci..

[14] Johnson T.J., Gupta K.M., Fabian J., Albright T.H., Kiser P.F. (2010). Segmented polyurethane intravaginal rings for the sustained combined delivery of antiretroviral agents dapivirine and tenofovir. Eur. J. Pharm. Sci..

[15] Verstraete G., Vandenbussche L., Kasmi S., Nuhn L., Brouckaert D., Van Renterghem J., Grymonpré W., Vanhoorne V., Coenye T., De Geest B.G. (2017). Thermoplastic polyurethane-based intravaginal rings for prophylaxis and treatment of (recurrent) bacterial vaginosis. Int. J. Pharm..

[16] Mesquita P.M.M., Rastogi R., Segarra T.J., Teller R.S., Torres N.M., Huber A.M., Kiser P.F., Herold B.C. (2012). Intravaginal ring delivery of tenofovir disoproxil fumarate for prevention of HIV and herpes simplex virus infection. J. Antimicrob. Chemother..

[17] Gupta K.M., Pearce S.M., Poursaid A.E., Aliyar H.A., Tresco P.A., Mitchnik M.A., Kiser P.F. (2008). Polyurethane Intravaginal Ring for Controlled Delivery of Dapivirine, a Nonnucleoside Reverse Transcriptase Inhibitor of HIV-1. J. Pharm. Sci..

[18] Traore Y.L., Chen Y., Bernier A.-M., Ho E.A. (2015). Impact of Hydroxychloroquine-Loaded Polyurethane Intravaginal Rings on Lactobacilli. Antimicrob. Agents Chemother..

[19] Smith J.M., Rastogi R., Teller R.S., Srinivasan P., Mesquita P.M.M., Nagaraja U., McNicholl J.M., Hendry R.M., Dinh C.T., Martin A. (2013). Intravaginal ring eluting tenofovir disoproxil fumarate completely protects macaques from multiple vaginal simian-HIV challenges. Proc. Natl. Acad. Sci. USA.

[20] Johnson T.J., Clark M.R., Albright T.H., Nebeker J.S., Tuitupou A.L., Clark J.T., Fabian J., McCabe R.T., Chandra N., Doncel G.F. (2012). A 90-day tenofovir reservoir intravaginal ring for mucosal HIV prophylaxis. Antimicrob. Agents Chemother..

[21] Keller M.J., Mesquita P.M., Marzinke M.A., Teller R., Espinoza L., Atrio J.M., Lo Y., Frank B., Srinivasan S., Fredricks D.N. (2016). A phase 1 randomized placebo-controlled safety and pharmacokinetic trial of a tenofovir disoproxil fumarate vaginal ring. AIDS.

[22] Teller R.S., Malaspina D.C., Rastogi R., Clark J.T., Szleifer I., Kiser P.F. (2016). Controlling the hydration rate of a hydrophilic matrix in the core of an intravaginal ring determines antiretroviral release. J. Control. Release.

[23] Smith J.M., Srinivasan P., Teller R.S., Lo Y., Dinh C.T., Kiser P.F., Herold B.C. (2015). Tenofovir disoproxil fumarate intravaginal ring protects high-dose depot medroxyprogesterone acetate-treated macaques from multiple SHIV exposures. J. Acquir. Immune Defic. Syndr..

[24] Clark M.R., Johnson T.J., McCabe R.T., Clark J.T., Tuitupou A., Elgendy H., Friend D.R., Kiser P.F. (2012). A hot-melt extruded intravaginal ring for the sustained delivery of the antiretroviral microbicide UC781. J. Pharm. Sci..

[25] Friend D.R., Clark J.T., Kiser P.F., Clark M.R. (2013). Multipurpose prevention technologies: Products in development. Antiviral Res..

[26] Claeys B., Bruyn S. De, Hansen L., Beer T. De, Remon J.P., Vervaet C. (2014). Release characteristics of polyurethane tablets containing dicarboxylic acids as release modifiers—A case study with diprophylline. Int. J. Pharm..

[27] Lazarus J., Pagliery M., Lachman L. (1964). Factors Influencing the Release of a Drug from a Prolonged-Action Matrix. J. Pharm. Sci..

[28] Anderson J.M., Hiltner A., Wiggins M.J., Schubert M.A., Collier T.O., Kao W.J., Mathur A.B. (1998). Recent advances in biomedical polyurethane biostability and biodegradation. Polym. Int..

[29] Cooper S.L., Guan J. (2016). Advances in Polyurethane Biomaterials.

[30] Boretos J.W., Pierce W.S. (1968). Segmented polyurethane: A polyether polymer. An initial evalution for biomedical applications. J. Biomed. Mater. Res..

[31] Gogolewski S. (1989). Selected topics in biomedical polyurethanes. A review. Colloid Polym. Sci..

[32] Gunatillake P.A., Martin D.J., Meijs G.F., McCarthy S.J., Adhikari R. (2003). Designing Biostable Polyurethane Elastomers for Biomedical Implants. Aust. J. Chem..

[33] Lamba N.M.K., Woodhouse K.A., Cooper S.L. (1997). Polyurethanes in Biomedical Applications.

[34] Vermette P., Griesser H.J., Laroche G., Guidoin R. (2001). Biomedical Applications of Polyurethanes.

[35] Irusta L., Fernandez-Berridi M.J. (2000). Aromatic poly(ester–urethanes): Effect of the polyol molecular weight on the photochemical behaviour. Polymer.

[36] DiBattista G., Peerlings H.W.I., Kaufhold W. (2003). Aliphatic TPUs for light-stable applications. Rubber World.

[37] Szycher M., Siciliano A.A. (1991). An Assessment of 2,4 TDA Formation from Surgitek Polyurethane Foam under Simulated Physiological Conditions. J. Biomater. Appl..

[38] Kääriä K., Hirvonen A., Norppa H., Piirilä P., Vainio H., Rosenberg C. (2001). Exposure to 4,4′-methylenediphenyl diisocyanate (MDI) during moulding of rigid polyurethane foam: Determination of airborne MDI and urinary 4,4′-methylenedianiline (MDA). Analyst.

[39] Jabbari E., Khakpour M. (2000). Morphology of and release behavior from porous polyurethane microspheres. Biomaterials.

[40] Thompson D.G., Osborn J.C., Kober E.M., Schoonover J.R. (2006). Effects of hydrolysis-induced molecular weight changes on the phase separation of a polyester polyurethane. Polym. Degrad. Stab..

[41] Saad B., Hirt T.D., Welti M., Uhlschmid G.K., Neuenschwander P., Suter U.W. (1997). Development of degradable polyesterurethanes for medical applications: In vitro and in vivo evaluations. J. Biomed. Mater. Res..

[42] Kaur M., Gupta K.M., Poursaid A.E., Karra P., Mahalingam A., Aliyar H.A., Kiser P.F. (2011). Engineering a degradable polyurethane intravaginal ring for sustained delivery of dapivirine. Drug Deliv. Transl. Res..

[43] Yu J., Lin F., Lin P., Gao Y., Becker M.L. (2014). Phenylalanine-Based Poly(ester urea): Synthesis, Characterization, and in vitro Degradation. Macromolecules.

[44] Rychlý J., Lattuati-Derieux A., Lavédrine B., Matisová-Rychlá L., Malíková M., Csomorová K., Janigová I. (2011). Assessing the progress of degradation in polyurethanes by chemiluminescence and thermal analysis. II. Flexible polyether- and polyester-type polyurethane foams. Polym. Degrad. Stab..

[45] Yilgör E., Burgaz E., Yurtsever E., Yilgör İ. (2000). Comparison of hydrogen bonding in polydimethylsiloxane and polyether based urethane and urea copolymers. Polymer.

[46] Wiggins M.J., Wilkoff B., Anderson J.M., Hiltner A. (2001). Biodegradation of polyether polyurethane inner insulation in bipolar pacemaker leads. J. Biomed. Mater. Res..

[47] Christenson E.M., Dadsetan M., Wiggins M., Anderson J.M., Hiltner A. (2004). Poly(carbonate urethane) and poly(ether urethane) biodegradation: In vivo studies. J. Biomed. Mater. Res. Part A.

[48] Ikeda Y., Kohjiya S., Takesako S., Yamashita S. (1990). Polyurethane elastomer with PEO-PTMO-PEO soft segment for sustained release of drugs. Biomaterials.

[49] Anderson J.M., Rodriguez A., Chang D.T. (2008). Foreign body reaction to biomaterials. Semin. Immunol..

[50] Kricheldorf H.R., Quirk R.P., Holden G. (2004). Thermoplastic Elastomers.

[51] Ahn T.O., Choi I.S., Jeong H.M., Cho K. (1993). Thermal and mechanical properties of thermoplastic polyurethane elastomers from different polymerization methods. Polym. Int..

[52] Arifin D.Y., Lee L.Y., Wang C.-H. (2006). Mathematical modeling and simulation of drug release from microspheres: Implications to drug delivery systems. Adv. Drug Deliv. Rev..

[53] Siepmann J., Siepmann F. (2008). Mathematical modeling of drug delivery. Int. J. Pharm..

[54] Clark J.T., Johnson T.J., Clark M.R., Nebeker J.S., Fabian J., Tuitupou A.L., Ponnapalli S., Smith E.M., Friend D.R., Kiser P.F. (2012). Quantitative evaluation of a hydrophilic matrix intravaginal ring for the sustained delivery of tenofovir. J. Control. Release.

[55] Ugaonkar S.R., Clark J.T., English L.B., Johnson T.J., Buckheit K.W., Bahde R.J., Appella D.H., Buckheit R.W., Kiser P.F. (2015). An Intravaginal Ring for the Simultaneous Delivery of an HIV-1 Maturation Inhibitor and Reverse-Transcriptase Inhibitor for Prophylaxis of HIV Transmission. J. Pharm. Sci..

[56] van Laarhoven J.A., Kruft M.A., Vromans H. (2002). In vitro release properties of etonogestrel and ethinyl estradiol from a contraceptive vaginal ring. Int. J. Pharm..

[57] Crank J. (1979). The Mathematics of Diffusion.

[58] Vergnaud J.-M. (1993). Controlled Drug Release of Oral Dosage Forms.

[59] Higuchi T. (1963). Mechanism of sustained-action medication. Theoretical analysis of rate of release of solid drugs dispersed in solid matrices. J. Pharm. Sci..

[60] Claeys B., Vervaeck A., Hillewaere X.K.D., Possemiers S., Hansen L., De Beer T., Remon J.P., Vervaet C. (2015). Thermoplastic polyurethanes for the manufacturing of highly dosed oral sustained release matrices via hot melt extrusion and injection molding. Eur. J. Pharm. Biopharm..

[61] Yasuda H., Lamaze C.E., Ikenberry L.D. (1968). Permeability of solutes through hydrated polymer membranes. Part I. Diffusion of sodium chloride. Die Makromol. Chem..

[62] Go¨pferich A., Langer R. (1995). Modeling monomer release from bioerodible polymers. J. Control. Release.

[63] Langer R., Peppas N. (1983). Chemical and Physical Structure of Polymers as Carriers for Controlled Release of Bioactive Agents: A Review. J. Macromol. Sci. Part C.

[64] Ritger P.L., Peppas N.A. (1987). A simple equation for description of solute release II. Fickian and anomalous release from swellable devices. J. Control. Release.

[65] Hafeman A.E., Li B., Yoshii T., Zienkiewicz K., Davidson J.M., Guelcher S.A. (2008). Injectable Biodegradable Polyurethane Scaffolds with Release of Platelet-derived Growth Factor for Tissue Repair and Regeneration. Pharm. Res..

[66] Hafeman A.E., Zienkiewicz K.J., Carney E., Litzner B., Stratton C., Wenke J.C., Guelcher S.A. (2010). Local Delivery of Tobramycin from Injectable Biodegradable Polyurethane Scaffolds. J. Biomater. Sci. Polym. Ed..

[67] Hombreiro-Pérez M., Siepmann J., Zinutti C., Lamprecht A., Ubrich N., Hoffman M., Bodmeier R., Maincent P. (2003). Non-degradable microparticles containing a hydrophilic and/or a lipophilic drug: Preparation, characterization and drug release modeling. J. Control. Release.

[68] Almeida A., Brabant L., Siepmann F., De Beer T., Bouquet W., Van Hoorebeke L., Siepmann J., Remon J.P., Vervaet C. (2012). Sustained release from hot-melt extruded matrices based on ethylene vinyl acetate and polyethylene oxide. Eur. J. Pharm. Biopharm..

[69] Tallury P., Alimohammadi N., Kalachandra S. (2007). Poly(ethylene-co-vinyl acetate) copolymer matrix for delivery of chlorhexidine and acyclovir drugs for use in the oral environment: Effect of drug combination, copolymer composition and coating on the drug release rate. Dent. Mater..

[70] Reddy T.T., Hadano M., Takahara A. (2006). Controlled Release of Model Drug from Biodegradable Segmented Polyurethane Ureas: Morphological and Structural Features. Macromol. Symp..

[71] Hsu T.T.-P., Langer R. (1985). Polymers for the controlled release of macromolecules: Effect of molecular weight of ethylene-vinyl acetate copolymer. J. Biomed. Mater. Res..

[72] Zhou L., Liang D., He X., Li J., Tan H., Li J., Fu Q., Gu Q. (2012). The degradation and biocompatibility of pH-sensitive biodegradable polyurethanes for intracellular multifunctional antitumor drug delivery. Biomaterials.

[73] Shoaib M., Bahadur A., Iqbal S., Rahman M.S.U., Ahmed S., Shabir G., Javaid M.A. (2017). Relationship of hard segment concentration in polyurethane-urea elastomers with mechanical, thermal and drug release properties. J. Drug Deliv. Sci. Technol..

[74] Verstraete G., Van Renterghem J., Van Bockstal P.J., Kasmi S., De Geest B.G., De Beer T., Remon J.P., Vervaet C. (2016). Hydrophilic thermoplastic polyurethanes for the manufacturing of highly dosed oral sustained release matrices via hot melt extrusion and injection molding. Int. J. Pharm..

[75] Kim J.-E., Kim S.-R., Lee S.-H., Lee C.-H., Kim D.-D. (2000). The effect of pore formers on the controlled release of cefadroxil from a polyurethane matrix. Int. J. Pharm..

[76] Donelli G., Francolini I., Ruggeri V., Guaglianone E., D’Ilario L., Piozzi A. (2006). Pore formers promoted release of an antifungal drug from functionalized polyurethanes to inhibit Candida colonization. J. Appl. Microbiol..

[77] Sreenivasan K. (2001). Effect of blending methyl β-cyclodextrin on the release of hydrophobic hydrocortisone into water from polyurethane. J. Appl. Polym. Sci..

[78] Langer R., Folkman J. (1976). Polymers for the sustained release of proteins and other macromolecules. Nature.

[79] Lindholm T., Lindholm B.-Å., Niskanen M., Koskiniemi J. (1986). Polysorbate 20 as a drug release regulator in ethyl cellulose film coatings. J. Pharm. Pharmacol..

[80] Bodmeier R., Paeratakul O. (1990). Theophylline Tablets Coated with Aqueous Latexes Containing Dispersed Pore Formers. J. Pharm. Sci..

[81] Frohoff-Hülsmann M.A., Schmitz A., Lippold B.C. (1999). Aqueous ethyl cellulose dispersions containing plasticizers of different water solubility and hydroxypropyl methylcellulose as coating material for diffusion pellets: I. Drug release rates from coated pellets. Int. J. Pharm..

[82] Sauer D., Watts A.B., Coots L.B., Zheng W.C., McGinity J.W. (2009). Influence of polymeric subcoats on the drug release properties of tablets powder-coated with pre-plasticized Eudragit® L 100-55. Int. J. Pharm..

[83] Irfan M., Ahmed A.R., Kolter K., Bodmeier R., Dashevskiy A. (2017). Curing mechanism of flexible aqueous polymeric coatings. Eur. J. Pharm. Biopharm..

[84] Bounds W., Szarewski A., Lowe D., Guillebaud J. (1993). Preliminary report of unexpected local reactions to a progestogen-releasing contraceptive vaginal ring. Eur. J. Obstet. Gynecol. Reprod. Biol..

[85] Koetsawang S., Gao J., Krishna U., Cuadros A., Dhall G.I., Wyss R., la Puenta J.R., Andrade A.T.L., Khan T., Kononova E.S. (1990). Microdose intravaginal levonorgestrel contraception: A multicentre clinical trial. Contraception.

[86] Morrow Guthrie K., Vargas S., Shaw J.G., Rosen R.K., van den Berg J.J., Kiser P.F., Buckheit K., Bregman D., Thompson L., Jensen K. (2015). The Promise of Intravaginal Rings for Prevention: User Perceptions of Biomechanical Properties and Implications for Prevention Product Development. PLoS One.

[87] Baum M.M., Butkyavichene I., Gilman J., Kennedy S., Kopin E., Malone A.M., Nguyen C., Smith T.J., Friend D.R., Clark M.R. (2012). An intravaginal ring for the simultaneous delivery of multiple drugs. J. Pharm. Sci..

[88] ASTM International (2015). ASTM D2240-15e1, Standard Test Method for Rubber Property—Durometer Hardness.

[89] ISO (2014). ISO 8009 Mechanical Contraceptives—Reusable Natural and Silicone Rubber Contraceptive Diaphragms—Requirements and Tests.

[90] Clark J.T., Clark M.R., Shelke N.B., Johnson T.J., Smith E.M., Andreasen A.K., Nebeker J.S., Fabian J., Friend D.R., Kiser P.F. (2014). Engineering a Segmented Dual-Reservoir Polyurethane Intravaginal Ring for Simultaneous Prevention of HIV Transmission and Unwanted Pregnancy. PLoS ONE.

[91] Crnich C.J., Halfmann J.A., Crone W.C., Maki D.G. (2005). The Effects of Prolonged Ethanol Exposure on the Mechanical Properties of Polyurethane and Silicone Catheters Used for Intravascular Access. Infect. Control Hosp. Epidemiol..

[92] Massey L.K. (2005). The Effects of Sterilization Methods on Plastics and Elastomers: The Definitive User’s Guide and Databook.

[93] Abraham G.A., Frontini P.M., Cuadrado T.R. (1997). Physical and mechanical behavior of sterilized biomedical segmented polyurethanes. J. Appl. Polym. Sci..

[94] Simmons A., Hyvarinen J., Poole-Warren L. (2006). The effect of sterilisation on a poly(dimethylsiloxane)/poly(hexamethylene oxide) mixed macrodiol-based polyurethane elastomer. Biomaterials.

[95] Gorna K., Gogolewski S. (2003). The effect of gamma radiation on molecular stability and mechanical properties of biodegradable polyurethanes for medical applications. Polym. Degrad. Stab..

[96] Ahmed M., Punshon G., Darbyshire A., Seifalian A.M. (2013). Effects of sterilization treatments on bulk and surface properties of nanocomposite biomaterials. J. Biomed. Mater. Res. Part B Appl. Biomater..

